# The modularization design and autonomous motion control of a new baby stroller

**DOI:** 10.3389/fnhum.2022.1000382

**Published:** 2022-09-30

**Authors:** Chunhong Zhang, Zhuoting He, Xiaotong He, Weifeng Shen, Lin Dong

**Affiliations:** ^1^School of Art and Design, University of Electronic Science and Technology of China, Zhongshan Institute, Zhongshan, China; ^2^Weihai Institute for Bionics, Jilin Univerisity, Weihai, China; ^3^College of Building Engineering, Xiamen City University, Xiamen, China; ^4^Center on Frontiers of Computing Studies, Peking University, Beijing, China

**Keywords:** motor control, modularization design, baby stroller, safety-enhanced, autonomous motion control, model predictive control (MPC)

## Abstract

The increasing number of newborns has stimulated the infant market. In particular, the baby stroller, serving as an important life partner for both babies and parents, has attracted more attention from society. Stroller design and functionality are of vital importance to babies' physiological and psychological health as well as brain development. Therefore, in this paper, we propose a modularization design method for the novel four-wheeled baby stroller based on the KANO model to ensure the mechanical safety and involve more functionalities. Manual control of the baby stroller requires the rapid response of human motor systems in a completely controlled manner, which could be a potential risk. To enhance the safety and stability of the stroller motion, especially in situations where manual control is hard to achieve (e.g., sharp turns), we propose an autonomous motion control scheme based on model predictive control. Both the modularization design and the motion controller are verified in the MATLAB simulation environment through path tracking tasks. The feasibility is validated by the satisfactory experimental results with lateral position error in a reasonable range and good trajectory smoothness.

## 1. Introduction

The past century has witnessed a global increase in the number of newborns, which has also stimulated the demand for baby products. The four-wheeled stroller, as a life partner for both newborn babies and parent groups, creates a convenient and worry-free lifestyle by empowering and assisting baby transportation and improving the quality of the parenting career, which explains why baby strollers are becoming increasingly popular among the younger generations of millennial parents (Lee et al., 2021). The baby stroller market is expected to be growing at a growth rate of 5.8% in the forecast period of 2022–2029 and is expected to reach the USD 3.88 billion by 2029, which shows that the stroller market outlook is very promising (GrandViewResearch, 2022). Nowadays, parents are getting more concerned about their children's safety when using strollers everywhere, which contributes greatly to the significance of baby stroller design and application research (Dols et al., 2013; Wang et al., 2021).

The stroller designs in history reveal different strengths and weaknesses, which have provided good references for the improvement of modern strollers. Despite the widespread attention and interest that the design of baby strollers has attracted in recent years, most existing stroller products tend to be directly designed by the mechanical structure according to the original demand, whose functions and the requirements that need to be fulfilled are still not satisfied and remain to be improved (Bao et al., 2016). Due to the continuous changes in the physiological functions of infants during the growth process, there are many details that need to be considered and paid attention to during product design. In terms of safety, there are many safety hazards in existing baby strollers. For example, the neglect of detailed parts results in active infants being vulnerable to scratches from exposed parts. As for the use of color, the designer did not adopt to combine the visual development characteristics of infants and the psychological perception of color, but arbitrarily used high brightness and high purity of bright colors, resulting in visual and psychological harm to infants (Ma and Zhou, 2016). Secondly, in the aspect of functionality, the functions of strollers on the market are too single in design and cannot better meet the needs of users in different periods. Moreover, in the whole design process of strollers, designers fail to fully refer to ergonomics to guide the design of baby stroller components, and neglect to analyze and study the design of baby strollers from the physiological characteristics and features of users, resulting in many incongruous effects between strollers and users (Veras, 2012; Sehat and Nirmal, 2017; Kamio and Aihara, 2020). Nowadays, as millennial parents have become the primary demographic for childbirth, more novel and personalized consumption consciousness and concepts have fueled the desire to shop. However, without considering its safety, functionality, design, and other important factors, young parents tend to base their shopping decisions on brands, prices or recommendations from friends when purchasing products, which has led to the emergence of some substandard products in the marketplace and caused adverse impacts on the development of the baby products market (Muraki, 2006; Garciaguirre et al., 2007; Azie Noor Marlia, 2015). As opposed to cookie-cutter stroller products, the design of baby strollers based on modularization is more in line with the needs of modern young parents, for it is able to provide more possibilities for stroller design. We investigate how the modular design can lead to a more sustainable future for the production and use of baby strollers. Past stroller research has seen continuous advances in stroller design.

The study of dynamic behavior and forces involved in the movement of baby strollers when on board public transportation have always been in the process of being pushed forward in order to guarantee the safety of strollers. Analysis of the forces transmitted to the baby strollers and their behavior during normal operation and critical maneuvers in public places have been focused on to address issues concerning the use of baby strollers (Son, 2010). To improve the performance of modern strollers so as to meet the daily needs of more families, experimental development of intelligent strollers equipped with an advanced electric hybrid assistance system that uses in-wheel-type motors is well underway. Taking into account the fact that a parent has to hold both the baby and its carriage while ascending or descending a flight of stairs, light-weight and easy-to-fold baby carriages are commonly used in Japan today (Kawashima, 2009).

With the ever-improving advancement of artificial intelligence (AI) technology, AI has been used in the fields of product design and assembly (Wu and Kilian, 2018; Chen et al., 2020). For example, Thomas et al. (2018) investigated autonomous robotic assembly based on reinforcement learning technology. Ren et al. (2017) used knowledge-based engineering (KBE) to set up the open product architecture of the baby stroller for design personalization. Tsai et al. (2007) proposed a computer-aided product color design with artificial intelligence within the product design cycle.

Apart from the novel design of the baby stroller, its mobility is also necessary in terms of market feasibility. Stability and safety are of vital importance for the motion control of the four-wheeled stroller. The longitudinal of the stroller can be directly controlled by the human user through the handbrake. Therefore, in this paper, we only concentrate on the lateral motion stability control. The key to the lateral motion stability control for the baby stroller lies in the trajectory tracking accuracy and smoothness. To achieve this purpose, researchers have proposed various lateral control approaches based on different techniques such as neural networks (Ho et al., 2012), fuzzy adaptive control (Onieva et al., 2011; Guo et al., 2013), PID (Proportion Integral Derivative) control (Wu et al., 2009; Han et al., 2017), optimal control (Gutjahr et al., 2016; Nahidi et al., 2017), etc. Inspired by the task decomposition principle, Ho et al. (2012) presented a novel fused feed-forward neural network controller which can be applied to control systems requiring manipulation of two input variables. Guo et al. (2013) proposed an adaptive fuzzy-sliding mode control strategy to deal with nonlinearities, parametric uncertainties and external disturbances, whose stability is proven through the Lyapunov theory. Han et al. (2017) proposed a neural network PID controller for the path tracking control based on an established vehicle dynamics model and a second-order vehicle steering control system model. Nahidi et al. (2017) proposed a multi-layered optimal control structure for the system modularity design. The high-level optimization process is used to determine the required longitudinal force and yaw moment adjustments, while the low-level controller is designed to optimally regulate torque at each wheel. Wang et al. (2016) introduced a robust H ∞ output-feedback based controller to deal with the problems of network-induced time delay and tire force saturation for the vehicle lateral motion control. Zhao et al. (2007) designed a slip angle estimation method based on vehicle kinematic and dynamic models and proposed a combining sliding mode controller for the system.

In spite of the numerous effective approaches, many of them are applied to high-speed vehicles. In fact, wheeled mobile manipulators used to assist human mobility, such as walkers, rollators, or strollers, must have specially designed motion controllers. Abeygunawardhana and Toshiyuki (2007) proposed an adaptive-based approach where the PD controller gains are changed adaptively to improve the stability of the trajectory motion of the two-wheeled mobile robot in limited working space. Rahim et al. (2018) presented an intelligent self-propelling baby stroller with obstacle avoidance. The authors leveraged Bluetooth wireless and ultrasonic technologies to safely handle the stroller movement. The work by Ibrahim et al. (2017) focuses on the safety of stroller autonomous movement. The authors proposed an RGB-D visual feedback controller for a differential drive attachable wheel system in response to the position and distance of a parent.

The main advantage of MPC is the fact that it allows the current timeslot to be optimized, while keeping future timeslots in account. This is achieved by optimizing a finite time-horizon, but only implementing the current timeslot and then optimizing again, repeatedly, thus differing from a linear-quadratic regulator. Also, MPC has the ability to anticipate future events and can take control actions accordingly. PID controllers do not have this predictive ability. With these benefits, MPC is an appropriate method for the lateral control of wheeled mobiles. MPC has been widely studied in the vehicle motion control (Ni et al., 2016; Wei et al., 2019; Czibere et al., 2021; Zhang et al., 2022). For the low-speed baby stroller control, there are few research concerning MPC. Therefore, programming a lateral stability tracking controller based on MPC scheme can ensure the safety and flexibility of motion and replace the manual control in the environment with sharp turns. We propose to employ the model predictive control strategy for the lateral stability control design of the four-wheeled baby stroller to achieve a safe, stable and smooth tracking performance.

In order to open up new growth space for baby stroller design, we introduce a modularization design method to create a novel four-wheeled baby stroller with safety-enhanced movement posture control to cater to multifunctional demands. Besides, we augment the intelligent functionality of the designed baby stroller by achieving semi-autonomous control for a better user experience. Indeed, the designed baby stroller can be manually controlled, as most commercial products in the market do. However, manual control of the baby stroller could solely be easily performed for straight-line movements on plain ground. For trajectories with sharp turns, manual control may lose stability and even result in dangerous situations. Therefore, to ensure the safety, stability, and flexibility of the baby stroller, we propose to implement a semi-autonomous motion controller for it. In real scenarios, the baby lying in the sleeping basket may move its body; the caregiver may hold the stroller handle and exert forces on it. These situations are unpredictable, leading to path deviations from the reference. To take into consideration the uncertain disturbances from the environment, we adopt an MPC-based lateral stability tracking controller for the baby stroller to achieve safe and smooth operation for the sharp turn movements. Finally, we verify its feasibility through simulation from the aspects of control smoothness and trajectory tracking accuracy. To the best of our knowledge, this paper is the first work applying MPC to the motion control of the baby stroller. The main contributions of this work are listed as follows:

1. We employed the KANO model to classify and prioritize the user's expectations. Based on the understanding and analysis of the structure and the function requirements of the baby stroller, we introduced the modularization design theory to break down the product into multiple modular components to satisfy the multifunctional demands of both parents and children. The designed baby stroller is endowed with baby cushion mode, sleeping basket mode, and baby walker mode as well.
2. The ability to anticipate future events and take control actions accordingly makes MPC a good method for the lateral control of the baby stroller. In order to guarantee the safety and flexibility of the designed baby stroller, especially in environments with sharp turns where human manual control is not easy and even dangerous, we proposed to implement an MPC-based lateral stability tracking control scheme to empower and assist the stroller mobility.
3. We designed and carried out three trajectory tracking tasks (straight line tracking, circle path tracking, and arbitrary path tracking) to verify the feasibility of both the mechanical design and the MPC-based controller of the baby stroller in the MATLAB simulation environment. A compared experiment with MPC, LQR, and Stanley shows that MPC controller has higher tracking accuracy on the novel designed stroller.

The remainder of the paper is organized as follows: Section 2 describes the novel modularization design of the baby stroller in detail and provides analyses of target users, consumer market, structures, materials, and discusses the relative applications. Section 3 discusses the kinematic and dynamic of the designed baby stroller, and then introduces the safety-enhanced lateral movement posture control based on MPC. Section 4 verifies the designed controller in simulation and the experimental results are shown. Section 5 concludes the paper and presents the future work.

## 2. Novel design of baby strollers

**Figure 1:**
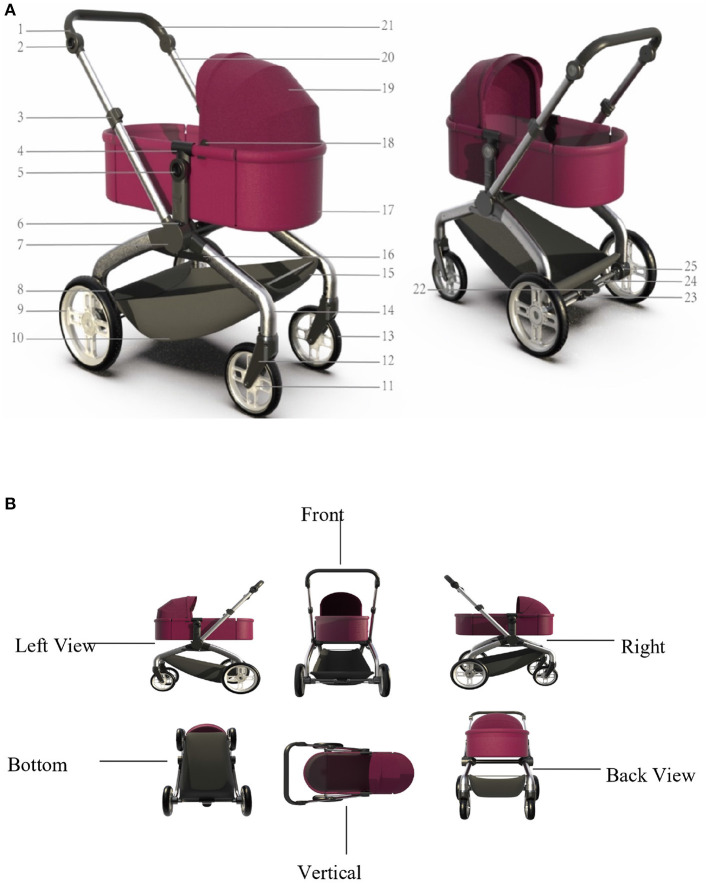
**(A)** Novel design of the baby stroller. 1. Handle rotation connection device; 2. Rotation button; 3. Pull button; 4. Sleeping basket fixing part; 5. Sleeping basket rotation button; 6. Handle rotation button; 7. Middle connector; 8. Rear wheel outer ring; 9. Rear wheel rim; 10. Storage basket; 11. Front wheel rim; 12. Front fork; 13. Front wheel outer ring; 14. Front foot tube; 15. Storage basket; 16. Aluminum strip; 17. Sleeping basket; 18. Awning fixing buckle; 19. Awning; 20. Aluminum tube; 21. Handle; 22. Brake pedal; 23. Brake control device; 24. Brake pipe; 25. Rear wheel fixing pipe; 26. Rear wheel fixing pipe. **(B)** Six views of the baby stroller.

This chapter is to present the design theory of modularization to the novel baby stroller, which focuses on the overview, categories and applications of the modularization design. Application of the modularization design in strollers, which includes the different stroller types and the features of them, will also be illustrated. Additionally, the main idea is to analyze the characteristics of the target users and the problems of using baby strollers in daily life. Hence the novel stroller design modes in which modularization is applied to construct the demand function will be introduced in detail. At present, there are many problems in the design of baby strollers, such as uneven quality, single function, poor portability, and lack of entertainment. By taking account of the safety, functionality, and portability, the optimized modularization design of the four-wheeled baby stroller is shown in Figures 1A,B. Figure 1A gives the elements of the modularization design in detail. Figure 1B shows the six views of the baby stroller.

### 2.1. Overview of modularization design

Based on the understanding and analysis of the structure as well as the function of the product, the modularization design is aimed to break down the product into multiple modular components. As each component is an independent unit with a corresponding function, modules can be combined and used interchangeably to form a product with new functions. Therefore, the modules are relatively independent and interconnected. There are three main features of the modularization design:

1. Relative independence. Each module component has an independent function and structure so that problems with one module do not affect the function and production of the others, and hence the producer is able to carry out targeted work in the production process.
2. Standardization. It refers to standardization of module interfaces, which is also characteristic of standardized mass production. Modularization of a product cannot be achieved if the product interfaces are inconsistent or the modules fail to connect to each other. Standardization can also define the structure and dimensions of the connectors of the product when the product is updated.
3. Functionality. The product consists of several modules, each with a different function, and multifunctionality is a feature of the product's modular design.

#### 2.1.1. Categories of modularization design

Modularization design is mainly divided into three categories and six basic types:

1. Interface modularization: It refers to the use of a standard interface through which modules are connected to each other in a certain location. Interface modularization can be divided into the following four types: shared type, replacement type, “tailor-made” type, and mixed type.
2. Bus-based modularization: It refers to the addition to the standard base components or the replacement of standardized modules with standardized interfaces, thus serving different purposes.
3. Combined modularization: It means that elements can be combined into new products or functions by standardizing the interfaces between modules in an arbitrary way. The same applies to building block toys.

#### 2.1.2. Cases of modularization design

1. ReTyer modular wheels (reTyre, 2022): It is a patented modular tyre system consisting of script tyres with integrated zips and removable skins. This allows the tread to be changed without the need to remove the tyres themselves. As a result, users can quickly and easily adapt Lintek's functions to their current route and achieve an optimal driving experience. This design is highly innovative and functional, greatly increasing agility and riding pleasure.
2. Ahooga modular bicycle (Ahooga, 2022): An electric compact cargo bike that is designed to be the ideal modern life companion. It has a high load capacity of 155 kg, including the possibility of carrying two children of all ages. Its “space saver” function makes it compatible with tight storage spaces in a short time and its modularity offers various configuration options: carbon belts, integrated gearbox, low-step sports handlebars. Transport of a wide range of goods, rack compatible with accessories such as baskets, side bags, and child seats, and the bike weighs only 16 kg.
3. Modular baby carrier (Kim and Yun, 2013): With its modular design, the baby carrier can be used in a variety of ways. The hip strap allows the child to be carried comfortably on the hip, and the hip seat can be converted to a carrying seat by means of fitting the rear unit. Further, by adding bottom accessories, it eventually acts as a complete baby carrier, which is even suitable for newborns. This product is characterized by its versatility and ease of handling, and most intentionally by its gender-neutral design.

### 2.2. Related research and analysis

#### 2.2.1. Analysis of target users

Physiological features: Babies in infancy have a peak in the first year of birth and growth. Weight and height increase rapidly in the first 3 months, and then the growth rate will slow down slightly. Most babies in the infant stage are in a sleep state and need to exercise with the assistance of adults to enhance their immunity. In the early stages of infancy, with strong curiosity, babies' ability to move their limbs begins to increase, and they gradually expand their range of activities (Türeyengil and Alppay, 2021).

Psychological characteristics: Infancy is the most important period of psychological development and personality growth in a child's life. The inner world is very rich, which is full of imagination and creativity (Lim, 2018). What's more, babies are very curious about the unknown and want to understand things in their own way.

(1) The period of establishing a sense of security: During the period from birth to crawling and walking, the most important thing is the infant's attachment to the mother. Because during this period, the baby can only explore the world through its eyes, mouth and touch, the mother's care and love are particularly important at this time. If the baby lacks maternal love, it is easy to lack the sense of trust. The baby establishes contact and trust with the outside world through the mother, so the baby can only establish a dependence on the mother and a sufficient sense of security when they feel enough attention and love (Parman and Edora, 2015).

(2) Independence and separation period: At the age of about 3 years old, children will begin to learn to be independent. During this process, they will continue to try and explore. Babies who were babbling before gradually began to express their emotions and needs in words. Then the child will be self-centered. At this stage, parents should not only encourage their children to move forward bravely, but also actively guide their values. If they dare not let their children try and contact the outside world at this stage, children will miss out on learning opportunities.

Zero to three years old is an important stage of children's psychological development. This period is the best period to cultivate children's psychological qualities and sound personalities, which will also play a key role in their future lives.

#### 2.2.2. Analysis of consumer market

Since the world has experienced sustained economic growth and rapid scientific and technological progress, as well as unprecedented and drastic demographic changes, according to the World Population Review, based in Walnut, California, USA, the global population has seen a rising trend year by year, which means the number of newborns in the world is also on the rise.

The upward trend in newborns leads to the fact that the stroller is the most commonly used product among baby products. The market size of the stroller industry is also increasing year by year. In today's world social environment, the demand for strollers has increased significantly (Sangameshwar et al., 2022). The baby stroller market is expected to be growing at a growth rate of 5.8% in the forecast period of 2022–2029 and is expected to reach USD 3.88 billion by 2029, which shows that the stroller market outlook is very promising.

#### 2.2.3. Analysis of structures and materials

Children's product design focuses on product safety. With diverse needs and individual differences in children, the structural design of baby strollers is the focus of the design (Xu and Shi, 2007).

Frame Structure: In the design of children's strollers, the frame structure is an important guarantee for the safety of the entire stroller. In production, the strength and hardness of the frame must be high enough. Second, in order to facilitate portability, weight and other factors, metal materials with light weight and high bearing capacity must be considered. Considering these two aspects, aluminum-magnesium alloy is generally used as the main material of the frame. Aluminum-magnesium alloy is light in weight, low in density, good in heat dissipation and strong in compression resistance, but it has the same strength and hardness as steel, and its weight is close to that of plastic. Some parts and connecting structures can be made of ABS engineering plastics, which are thermoplastics with good comprehensive properties. ABS plastic is strong, lightweight, hard in surface, very smooth, and easy to clean.

Seat: A seat determines the quality of a stroller. As it is directly in contact with the baby's skin, it is of great significance to choose the seat material with good air permeability. The most commonly used materials are polyester and Oxford cloth, which have good flame redundancy and breathability.

### 2.3. Application of modular design in baby strollers

#### 2.3.1. Versatrax multifunctional stroller

As strollers play a key role in parental mobility, they reflect the needs of both children and parents. Against this background, the design of strollers has realized the concept of their multifunctionality. Based on modularization fully adapted to children's lives, it offers four easy-to-set modes as well as a fast folding method. Furthermore, it is compatible with carrying strollers and baby carriers, while sport seats can be rotated as needed, or rearward facing back to the parent. Thanks to the cleverly designed folding mechanism, the stroller folds quickly and intuitively regardless of whether the seat is facing forward or backward.

#### 2.3.2. Quinny hubb duo modular stroller

The product is a spacious and comfortable modular stroller, which is tailored to the needs of parents. The stroller has an effortless fit because of its modular frame with various slots. For example, a stroller and a full-size handcart or one or two full-size seats can be positioned to face either the front or the back. The large shopping basket is easily accessible and can hold up to 20 kg of items. With a peek window above the body, the narrow foot-print makes the stroller ideal for operation in urban environments.

### 2.4. Four modes of the baby stroller

After investigation, we found that the existing stroller folding steps are complicated, which makes it not able to achieve fast folding. With a short lifespan and a single function, idle strollers make it difficult for users to place them. In view of the above issues, we adopt the modularization design method to combine the functions of a baby stroller, a walking walker and a picnic cushion.

Through the analysis of the user demand through the KANO model, the user's expectations and the corresponding modular design elements can be clarified. The KANO model is a screening tool invented by Professor Kano Noriaki of Tokyo University of Science to classify and prioritize the needs of users. It is based on analyzing the impact of user needs on their satisfaction. According to the relationship between different types of quality characteristics and customer satisfaction, the KANO model divides the quality characteristics of products and services into five categories: Must-Quality, One-dimensional Quality, Attractive Quality, Indifferent Quality, and Reverse Quality. Must-Quality refers to the elements that users consider essential to the product and without which the product is rendered unusable. One-dimensional Quality means that if the product has this function, it will be more perfect. One-dimensional Quality is accompanied by product quality and has a linear relationship with user satisfaction. Attractive Quality refers to the unexpected functions of users. No matter what kind of attractive quality is provided in the design process, it will give users an excellent user experience. If the product lacks such features, it will not affect the user's satisfaction. Indifferent Quality refers to the function points that users do not pay attention to. Increasing or decreasing this function will not affect user's satisfaction. Reverse Quality refers to the function points that users are more disgusted with, and its appearance will reduce user's satisfaction.

**Figure 2:**
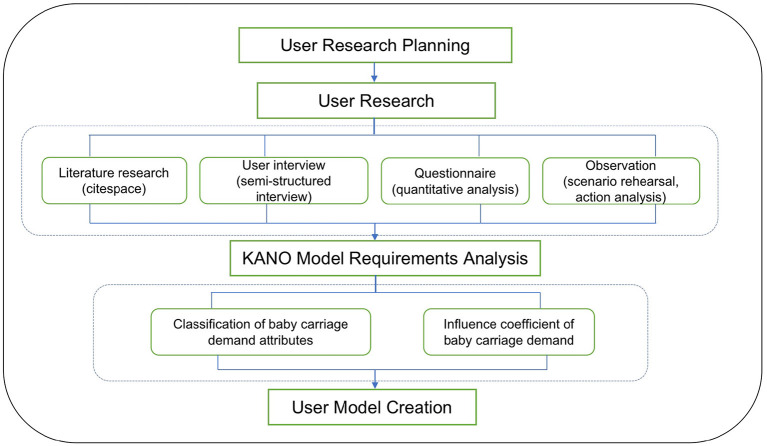
Process of user research on the baby stroller design.

After applying the tools of literature survey, market research, questionnaires, interviews, observations, and other methods to obtain user needs, we further employ the KANO model to classify the type of the requirements and prioritize the user's expectations. Through the analysis of the impact of user needs on their satisfaction, it reflects the nonlinear relationship between product performance and user's satisfaction which helps designers filter users' needs. Comparing all the collected Kano attribute questionnaires with the Kano model analysis table, we obtain the Kano category membership of demand indicators in questionnaires filled out by each user. After the analysis of the user demand through the KANO model, the user's expectations are classified and the corresponding modular design elements can further be clarified. On the basis of ensuring that a stroller has its basic function, it is endowed with the functions of a walker and a picnic cushion, which are to be combined with the current social environment of advocating green ideas. Figure 2 illustrates the process of user research on the baby chair design based on the KANO model.

**Figure 3:**
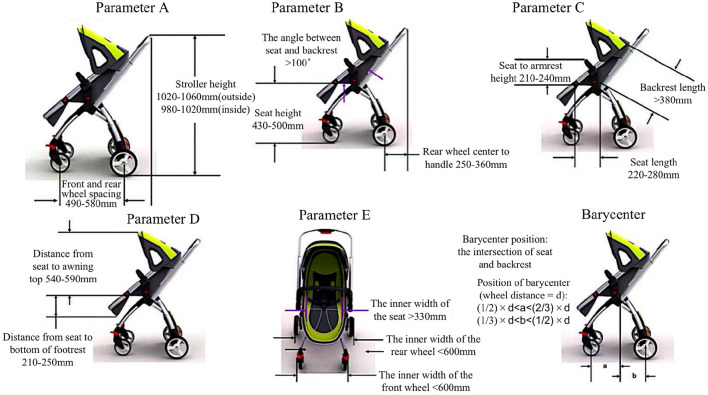
Standard size range of the baby stroller.

The dimensions of baby strollers are required to be designed in a strict range, and the size of the stroller can easily have an impact on the user's experience, so the design of the pram should comply with the regulations of the size range of the stroller in order to design an ergonomic product. The standard size range is shown in Figure 3.

**Figure 4:**
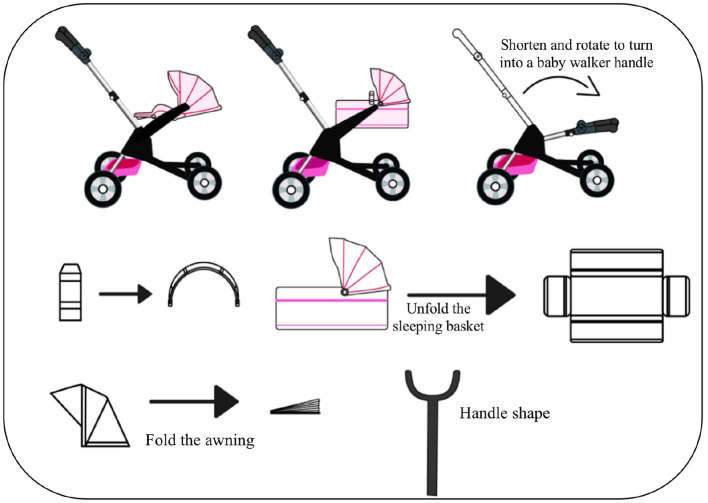
Detailed sketch.

Finally, one of the most feasible solutions was screened from dozens of preliminary sketch plans and a detailed sketch was drawn as shown in Figure 4. Because this solution is simple in shape-designing, minimalist in structure and more functional, it meets the different needs of users. Compared with other sketches, the stroller with this shape has a stronger landing ability, so this scheme was finally chosen for optimization.

**Figure 5:**
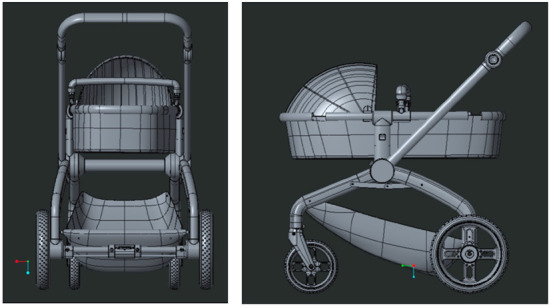
Preliminary draft model with Creo parametric.

In the 3D modeling of the design, Creo Parametric is used to construct the preliminary draft model. After the scheme is determined, Creo is used to refine the rest of the modeling and structural models. After completing the model establishment and assembly of each part, the overall modeling of the stroller is accomplished, and the core of gravity of the car is placed at the center of the car, so as to ensure the stability of the sleeping basket and the car body. The effect is as in Figure 5.

The following are the four modes of the modular baby stroller we designed, including baby cushion mode, sleeping basket mode, baby walker mode, and baby stroller mode.

#### 2.4.1. Baby cushion mode

The baby cushion mode is considered and designed for the hygienic problems of the baby outdoors. The sleeping basket is a very familiar and relatively hygienic environment for the baby, and the familiar taste and touch can make the baby feel a little more secure in the new environment. The cushion is formed by the unzipping of the sleeping basket, and the soft-fitting connection makes it more convenient for parents to switch functions. It can be unfolded and dried during cleaning and disinfection, which is more hygienic.

#### 2.4.2. Sleeping basket mode

The sleeping basket mode is suitable for infants of a young age. The sleeping basket can be taken out separately for travel without the whole car being pushed out of the door. This mode is convenient for parents to travel with their babies. The sleeping basket has a button that is easy to operate for disassembly and suitable for the novice father and mother.

#### 2.4.3. Baby walker mode

**Figure 6:**
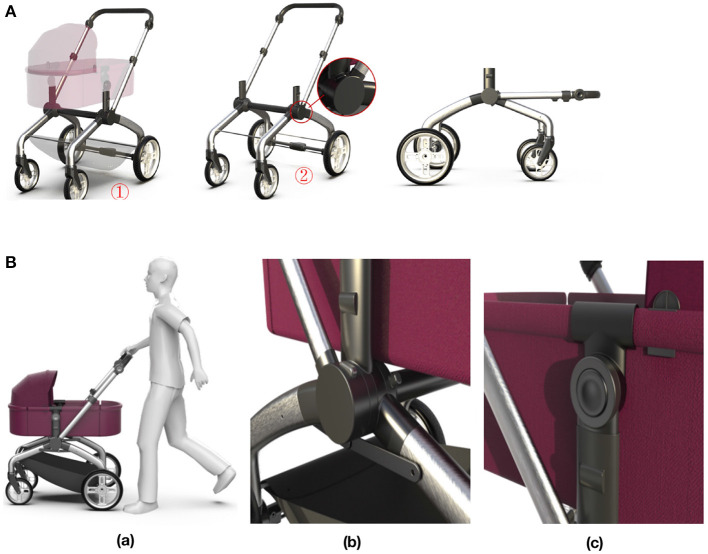
**(A)** Baby walker mode. ① Remove the storage basket and sleeping basket. ② Press the rotating button of the rotating handle to adjust to the appropriate position. **(B)** Baby stroller mode. **(a)** Baby stroller mode. **(b)** Middle connecting device. **(c)** Sleeping basket connector.

The walker mode (see in Figure 6A) is more suitable for infants around 12 months, that is, the toddler period. As an armrest support for infants, without adding too many parts and complicated conversion steps, the structure of the armrest can be shortened and rotated to a height suitable for a baby of 12 months.

#### 2.4.4. Baby stroller mode

The stroller mode (see in Figure 6B) is the most basic and longest-used function, which is suitable for babies aged 0–36 months. The stroller mode is convenient for the baby to go out, and its frame has a folding function, which provides convenience for the storage and travel of the car body. The middle connecting device (see in Figure 6Bb) can adjust the rotation angle of the handle according to the user's demand, so as to realize the body storage and the rotation of the handle. The sleeping basket connector (see in Figure 6Bc) allows the angle of the basket to be rotated by pressing the rotation button to further adjust the baby's sitting and sleeping position.

## 3. Safety-enhanced movement posture control

Safety is of vital importance to the design and control of the baby stroller. The manual control of the baby stroller is easy when the trajectory is a purely straight line. However, manual control becomes difficult and potentially dangerous for motions with sharp turns. Therefore, in this section, we implement a tracking controller based on the model predictive control algorithm and verify the performance of the four-wheeled stroller in the simulation environment.

### 3.1. Kinematic model of the baby stroller

**Figure 7:**
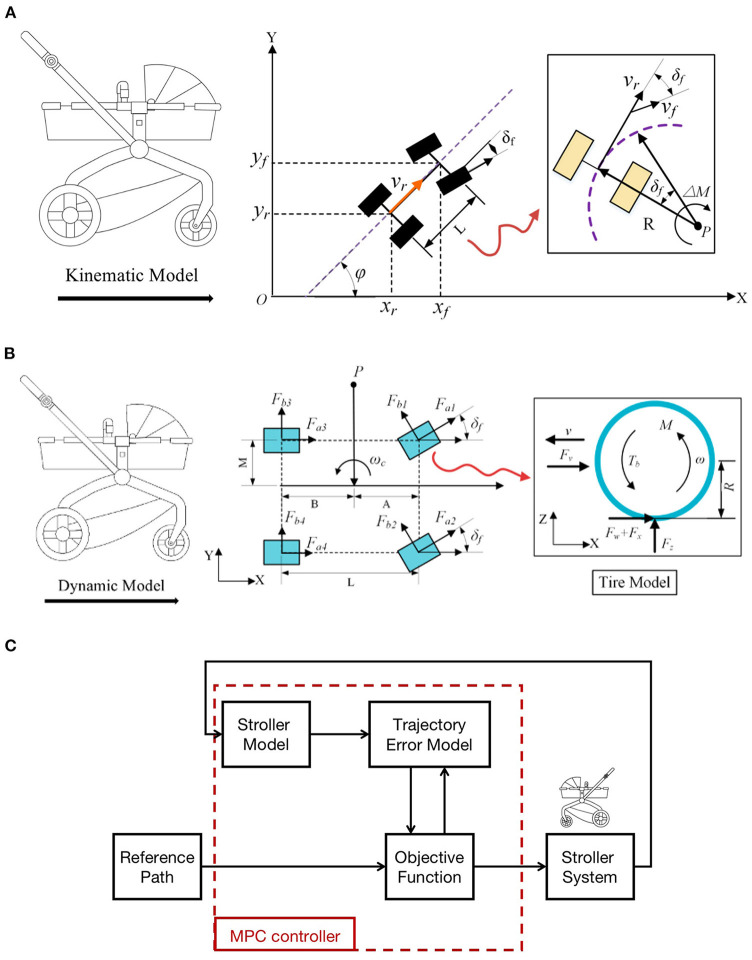
**(A)** Kinematic model of the four-wheeled baby stroller. **(B)** Dynamic model of the four-wheeled baby stroller and the tire model. **(C)** The MPC control diagram for the baby stroller.

Figure 7A shows the kinematic model of the baby stroller which is considered as a bicycle model.

The baby stroller is assumed to have planar motion, and the fact that only the front wheel can be steered is under consideration as well. The motion of the stroller is described as:

(1) $$\begin{array}{l} \begin{array}{l} {\dot{x}}_{f}sin\left(\varphi +\delta_{f}\;\right)-{\dot{y}}_{f}cos\left(\varphi +\delta_{f}\;\right)=0,\\ {\dot{x}}_{r}cos\varphi \;-{\dot{y}}_{r}sin\varphi =0, \end{array} \end{array}$$

where (*x*_*f*_, *y*_*f*_), (*x*_*r*_, *y*_*r*_) are the center coordinates of the location of the front and rear wheel axes while φ is the course angle describing the orientation of the stroller. δ_*f*_ is the steering angle of the front wheels.

From Equation (1), we define:

(2) $$\begin{array}{l} \begin{array}{l} {\dot{x}}_{r}\;=\;v_{r}cos\varphi ,\\ {\dot{y}}_{r}\;=\;v_{r}sin\varphi . \end{array} \end{array}$$

The angular velocity of the stroller ω is:

(3) $$\begin{array}{l} \omega \;=\;\frac{v_{r}tan\delta_{f}}{L}, \end{array}$$

where *L* is the wheelbase of the stroller.

Combining Equations (2) and (3), we write the kinematic model of the baby stroller as:

(4) $$\begin{array}{l} \left[\begin{array}{c} {\dot{x}}_{r}\\ {\dot{y}}_{r}\\ \dot{\varphi } \end{array}\right]=\;\left[\begin{array}{c} cos\varphi \\ sin\varphi \\ 0 \end{array}\right]v_{r}+\left[\begin{array}{c} 0\\ 0\\ 1 \end{array}\right]\omega . \end{array}$$

$\chi ={\left[{\dot{x}}_{r},{\dot{y}}_{r},\dot{\varphi }\right]}^{T}$ are the system states while $u={\left[v_{r},\omega \right]}^{T}$ are the control inputs.

### 3.2. Dynamic model of the baby stroller and tire model

Figure 7B shows the dynamic model of the four-wheeled baby stroller and the tire model. We still consider the bicycle model for the stroller dynamics. Note that Figure 7B is a conceptual model for the sake of illustration.

Let point *P* be the center of rotation of the stroller. Applying Newton's second law for longitudinal, lateral, and transverse motions (Gong et al., 2014; Shen et al., 2018; Jiang and Yang, 2019), we write the equations governing the system:

(5) $$\begin{array}{l} m(\ddot{x}-\dot{y}\omega_{c})=F_{a1}cos\delta_{f}+F_{a2}cos\delta_{f}+F_{a3}+F_{a4},\\ m(\ddot{y}+\dot{x}\omega_{c})=F_{b1}cos\delta_{f}+F_{b2}cos\delta_{f}+F_{b3}+F_{b4},\\ I_{z}\ddot{\varphi }=A(F_{b1}cos\delta_{f}+F_{b2}cos\delta_{f})-B(F_{b3}+F_{b4})\\ +M(-F_{a1}cos\delta_{f}+F_{a2}cos\delta_{f}-F_{a3}+F_{a4}), \end{array}$$

where *F*_*ai*_, *F*_*bi*_, *i* = 1, 2, 3, 4 are the longitudinal and lateral tire forces of the front and rear wheels, respectively; ω_*c*_ is the yaw velocity of the stroller center; *A* and *B* are the distances of the front and the rear tires from the stroller center.

Slip ratio plays an important role for the tire maintaining stable motion. The tire model, according to Figure 7B, can be described as:

(6) $$\begin{array}{l} \begin{array}{l} J\dot{\omega }=RF_{x}-RF_{\omega }-T_{b},\\ M\dot{v}=-F_{x}-\mu F_{v}, \end{array} \end{array}$$

where *J* and *M* are the stroller inertia and mass, respectively, *R* is the tire radius, μ is the adhesion coefficient. *F*_*x*_, *F*_*v*_, *F*_ω_, *T*_*b*_ denote the friction, air resistance, rolling resistance and braking torque, respectively. *F*_*x*_ is further defined as:

(7) $$\begin{array}{l} F_{x}=\mu F_{z}. \end{array}$$

The slip rate *S* is defined as:

(8) $$\begin{array}{l} S\;=\;\frac{v-\omega R}{v}. \end{array}$$

Now we choose the system states $x_{1}=\;\frac{v}{R},\;x_{2}=\omega ,x_{3}=\;S$, the tire function is rewritten as:

(9) $$\begin{array}{c} {\dot{x}}_{1}=-\frac{F_{v}+\mu F_{z}}{MR},\\ {\dot{x}}_{2}=\frac{\mu F_{z}R-F_{\omega }R-T_{b}}{J},\\ {\dot{x}}_{3}=\frac{1}{v}\left[\frac{\left(S-1\right)(F_{v}+\mu F_{z}+F_{z})}{M}+\frac{F_{z}R^{2}\left(T_{b}-\mu \right)}{J}\right]. \end{array}$$

### 3.3. MPC controller development for the baby stroller

The control diagram based on MPC for the baby stroller is presented in Figure 7C. Basically, the MPC algorithm is composed of three parts: a system model, system constraints and an objective function for optimization (Li et al., 2015b). The system model mathematically describes the behavior of the stroller in terms of the error of lateral trajectory tracking. System constraints consist of stroller actuator constraints, constraints for control smoothness as well as stability. The objective function is selected to minimize the tracking error and the control inputs based on stability and rapidity. We will present the three parts in detail in the following.

#### 3.3.1. Trajectory error model

Because the baby stroller's motion is slow, we derive the trajectory error model from the kinematic model rather than the complex dynamic model. According to Equation (4), we further write the stroller control system as:

(10) $$\begin{array}{l} \dot{\chi }=W\left(\chi ,u\right), \end{array}$$

where χ(*x, y*, φ) is the system state variable and *u*(*v*, δ) is the input variable.

The equation of the desired motion trajectory is:

(11) $$\begin{array}{l} {\dot{\chi }}_{d}=W\left(\chi_{d},u_{d}\right), \end{array}$$

with χ_*d*_, *u*_*d*_ being the desired state and input variables respectively.

Expanding Equation (10) at the desired trajectory using Taylor formulation and ignoring the high-order terms, we get the lateral error equations:

(12) $$\begin{array}{l} \begin{array}{l} {\dot{x}}_{e}=-v_{d}sin\varphi_{d}x_{e}+cos\varphi_{d}v_{e},\\ {\dot{y}}_{e}=v_{d}cos\varphi_{d}y_{e}+sin\varphi_{d}v_{e},\\ {\dot{\varphi }}_{e}=\frac{tan\delta_{d}}{L}v_{e}+\frac{v_{d}}{L\cos^{2}\delta_{d}}\delta_{e}, \end{array} \end{array}$$

where the subscript *e* denotes the error between the actual and the desired values, namely, $\chi_{e}=\left[\begin{array}{c} x-x_{d}\\ y-y_{d}\\ z-z_{d} \end{array}\right],u_{e}=\left[\begin{array}{c} v-v_{d}\\ \delta -\delta_{d} \end{array}\right]$.

We further discretize Equation (12) with a sampling interval *T* and express in a concise matrix form:

(13) $$\begin{array}{l} \begin{array}{l} \chi_{e}\left(k+1\right)=A\chi_{e}\left(k\right)+Bu_{e}\left(k\right),\\ A=\left[\begin{array}{ccc} 1 & 0 & -vTsin\varphi \\ 0 & 1 & vTcos\varphi \\ 0 & 0 & 1 \end{array}\right],\\ B=\left[\begin{array}{cc} Tcos\varphi & 0\\ Tsin\varphi & 0\\ \frac{tan\delta }{L}T & \frac{v}{L\cos^{2}\delta }T \end{array}\right]. \end{array} \end{array}$$

The observation function is defined as:

(14) $$\begin{array}{l} y\left(k\right)=C\chi_{e}\left(k\right). \end{array}$$

where *C* is an identity matrix.

#### 3.3.2. Objective function for optimization

The stroller should be able to track the desired reference trajectory in a rapid and safe manner, therefore, we design the objective optimization function as follows:

(15) $$\begin{array}{l} L\left(k\right)=\overset{N}{\underset{n=1}{\sum }}{\chi_{e}}^{T}\left(k+n|k\right)Q\chi_{e}\left(k+n|k\right)+{u_{e}}^{T}\left(k+n-1|k\right)Ru_{e}\left(k+n-1|k\right), \end{array}$$

where *Q* and *R* are the positive definite weighting matrices.

The first term of this objective function aims to let the stroller follow the desired trajectory with minimal tracking error and the second term reflects the control variable constraints. For better handling the control increment, we group χ_*e*_ and *u*_*e*_ to get a new state:

(16) $$\begin{array}{l} \xi \left(k\right)={\left[\chi_{e}\left(k\right),u_{e}\left(k-1\right)\right]}^{T}, \end{array}$$

which makes the new state function and observation function as:

(17) $$\begin{array}{l} \begin{array}{l} \xi \left(k+1\right)=A\xi \left(k\right)+B\Delta u_{e}\left(k\right),\\ \eta \left(k\right)=C\xi \left(k\right), \end{array} \end{array}$$

$A=\left[\begin{array}{cc} A & B\\ 0 & I_{N_{u}} \end{array}\right],B=\left[\begin{array}{c} B\\ I_{N_{u}} \end{array}\right],C=\left[\begin{array}{cc} I_{N_{x}} & 0 \end{array}\right]$, *I*_*N*_*u*__ and *I*_*N*_*x*__ are identity matrices with dimensions *N*_*u*_ and *N*_*x*_.

For prediction horizon *N*_*P*_ and control horizon *N*_*C*_, the model prediction output is:

(18) $$\begin{array}{l} Y=\psi \xi \left(k\right)+\Theta \Delta U, \end{array}$$

$Y=\left[\begin{array}{c} \eta \left(k+1\right)\\ \vdots \\ \eta \left(k+N_{P}\right) \end{array}\right],\psi =\left[\begin{array}{c} CA\\ \vdots \\ CA^{N_{P}} \end{array}\right],\Theta =\left[\begin{array}{ccc} CB & \cdots & 0\\ \vdots & \ddots & \vdots \\ CA^{N_{P}-1}B & \cdots & CA^{N_{P}-N_{C}}B \end{array}\right],\Delta U=\left[\begin{array}{c} \Delta u_{e}\left(k\right)\\ \vdots \\ \Delta u_{e}\left(k+N_{C}-1\right) \end{array}\right]$.

Substituting Equations (18) to (15), we can obtain a new quadratic objective optimization function:

(19) $$\begin{array}{l} \underset{\Delta U}{\text{min}}L=\frac{1}{2}\Delta U^{T}H\Delta U+g^{T}\Delta U, \end{array}$$

$H=\Theta^{T}\left(I_{N_{P}}⊗Q\right)\Theta +I_{N_{P}}⊗R,g={\left(\psi \xi \left(k\right)\right)}^{T}\left(I_{N_{P}}⊗Q\right)\Theta$, ⊗ denotes the Kronecker Product.

#### 3.3.3. Control constraints

To ensure the safety and stability of the baby stroller, it is required to bound the control input as well as the control increment during the optimization process.

For the control horizon *N*_*C*_, we have:

(20) $$\begin{array}{l} U=U_{t}+H_{I}\Delta U_{t}, \end{array}$$

$U=\left[\begin{array}{c} u\left(k\right)\\ \vdots \\ u\left(k+N_{C}-1\right) \end{array}\right],U_{t}=\left[\begin{array}{c} u\left(k-1\right)\\ \vdots \\ u\left(k-1\right) \end{array}\right],H_{I}=\left[\begin{array}{ccc} I_{N_{u}} & \cdots & 0\\ \vdots & \ddots & \vdots \\ I_{N_{u}} & \cdots & I_{N_{u}} \end{array}\right],\Delta U_{t}=\left[\begin{array}{c} \Delta u\left(k\right)\\ \vdots \\ \Delta u\left(k+N_{C}-1\right) \end{array}\right]$.

At each time step *i* = 0, 1, …, *k* + *N*_*C*_ − 1, we have the following control limit:

(21) $$\begin{array}{l} u_{min}\left(k+i\right)\leq u\left(k+i\right)\leq u_{max}\left(k+i\right). \end{array}$$

Therefore, we can write:

(22) $$\begin{array}{l} U_{min}\leq U_{t}+H_{I}\Delta U_{t}\leq U_{max}, \end{array}$$

which can be further transformed as:

(23) $$\begin{array}{l} \{\begin{array}{l} H_{I}\Delta U_{t}\leq U_{max}-U_{t},\\ -H_{I}\Delta U_{t}\leq -U_{min}+U_{t}. \end{array} \end{array}$$

Besides, the control increment limit is expressed as:

(24) $$\begin{array}{l} {\Delta U}_{min}\leq \Delta U_{t}\leq {\Delta U}_{max}. \end{array}$$

Equations (23) and (24) together are the final constraints for the optimization function Equation (19).

## 4. Results and discussion

To verify the performance of the MPC controller developed in the previous section, we designed three simulation experiments in MATLAB: one straight line tracking task, one circle path following task and one arbitrary path tracking task. The person who pushes the baby stroller could be the parents or grandparents, which means that the motion velocity for the semi-autonomous control is required to satisfy different situations. Therefore, in the three tasks, we set the desired longitudinal velocity to different values. All of the three tasks represent common scenarios in real life where manual control is not easy due to the initial position of the baby stroller or due to the large trajectory curvature of the turns.

### 4.1. The experimental results of straight line tracking task

**Figure 8:**
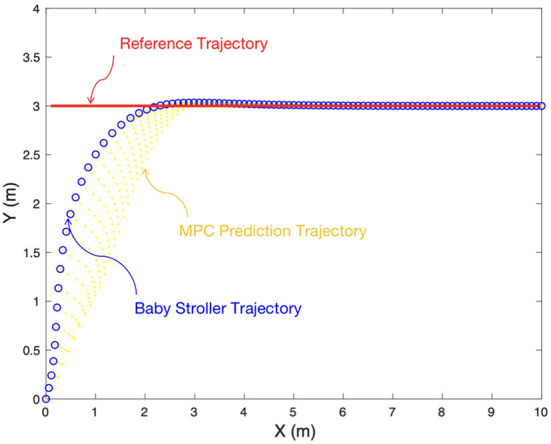
Experimental results of tracking the straight line reference.

**Figure 9:**
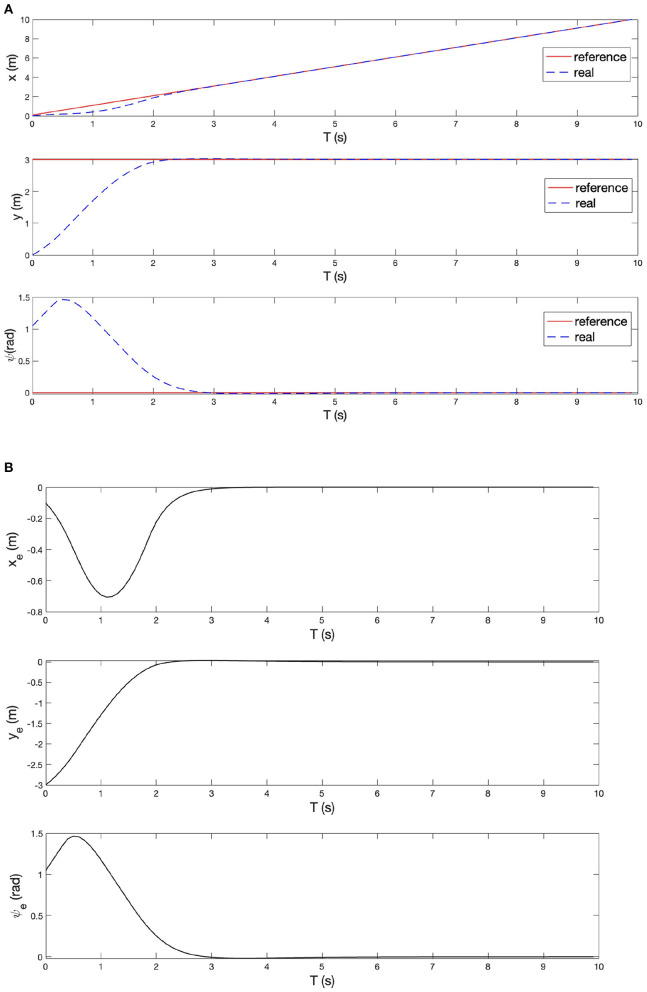
**(A)** Longitudinal position, lateral position, and yaw angle of the reference trajectory and of the baby stroller. **(B)** Longitudinal error, lateral error, and yaw angle error between the baby stroller and the reference trajectory.

**Figure 10:**
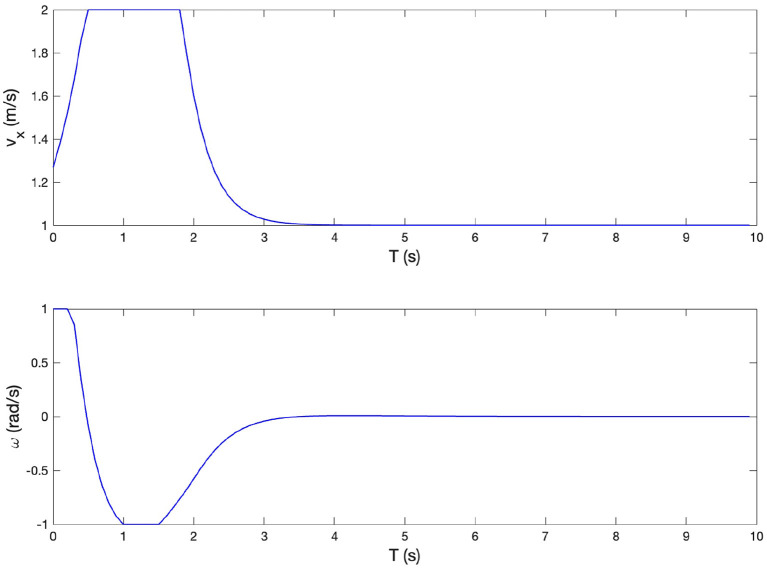
The longitudinal velocity and yaw velocity of the baby stroller.

The straight line tracking task requires that the baby stroller tracks the given reference trajectory, which is a straight line with a constant y position (*y* = 3 *m*) in the global frame, with a desired longitudinal velocity (*v* = 1 *m*/*s*). The stroller starts from the global origin with a yaw angle π/3. The simulation lasts 10 *s* with a sampling time interval of 0.1 *s*. The simulation process can be divided into four sequential sections: reference trajectory generation, variable initialization, system model establishment and controller implement. Figures 8–10 give the simulation results.

Figure 8 shows the desired reference trajectory (red solid lines), the MPC prediction trajectory (yellow dots) and the baby stroller trajectory (blue circles). It can be seen that the stroller trajectory quickly converges to the reference trajectory in <3 *s* and has a negligible tracking error after 3 *s*, leading to a satisfying control performance. The tracking performance is further demonstrated in Figures 9A,B in terms of system states. Figure 9A shows the longitudinal position, lateral position and yaw angle of the reference trajectory (red solid lines) and those of the baby stroller (blue dotted lines). Figure 9B shows the corresponding errors between the baby stroller and the reference trajectory. The curves are all smooth with little fluctuation, supporting the stability of the four-wheeled stroller. Furthermore, Figure 10 shows the control input, namely, the longitudinal velocity and yaw velocity of the stroller. The control is within the constraint range, proving the safety of the baby stroller.

### 4.2. The experimental results of circle path following task

**Figure 11:**
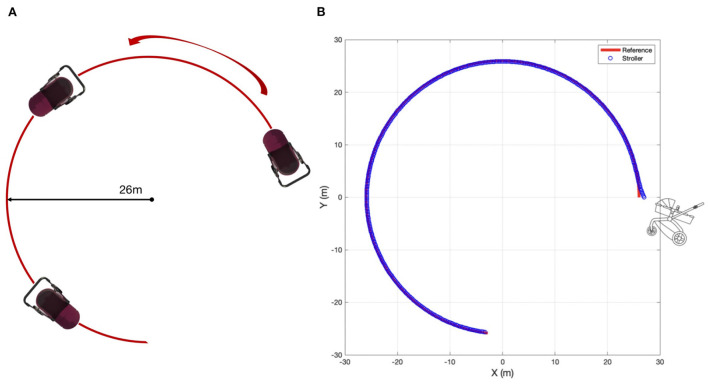
**(A)** The illustration of experimental setup for the circle path tracking task for the baby stroller control. **(B)** Experimental results of following the circle path.

**Figure 12:**
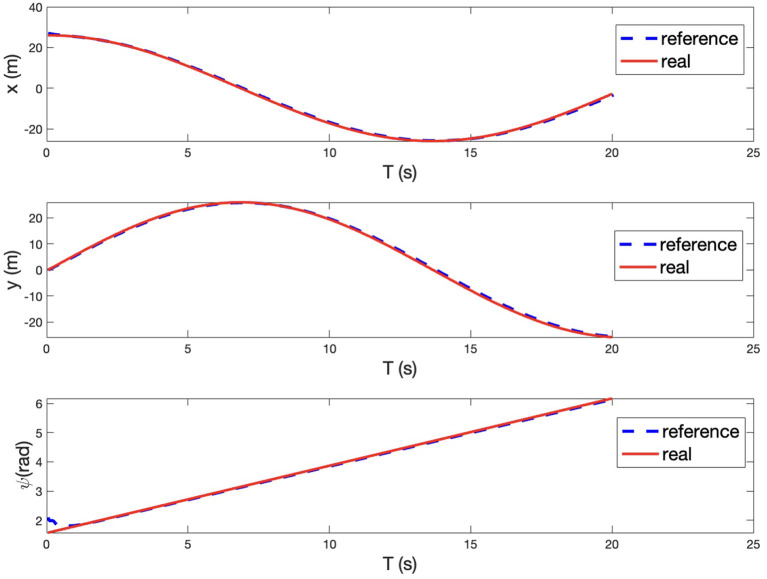
Longitudinal position, lateral position, and yaw angle of the reference trajectory and of the baby stroller when following the circle path.

To further demonstrate the controller effectiveness and the accuracy of the proposed MPC algorithm, a circle path following task is carried out in simulation with MATLAB. The experimental setup is illustrated in Figure 11. The reference path is a 26 m radius circle. The desired velocity is set at 6 *m*/*s*. The stroller starts from (27, 0) in the global frame with an initial yaw angle 2 ∗ π/3. The tracking performance is shown in Figure 11, where the red curve denotes the reference path while the blue circles are the stroller trajectory. From the co-simulation results, we observe that the proposed MPC-based control algorithm tracks the reference path well, with acceptable accuracy and smoothness, as reflected by the longitudinal, lateral positional and yaw angle tracking errors shown in Figure 12. Therefore, we can conclude that the MPC strategy can be used to improve the efficiency of the four-wheeled baby stroller. From Figure 12, we see that even though the trajectory curvature is large, indicating a sharp turn, our proposed controller still has good performance with satisfying tracking accuracy.

### 4.3. The experimental results of arbitrary path tracking task

**Figure 13:**
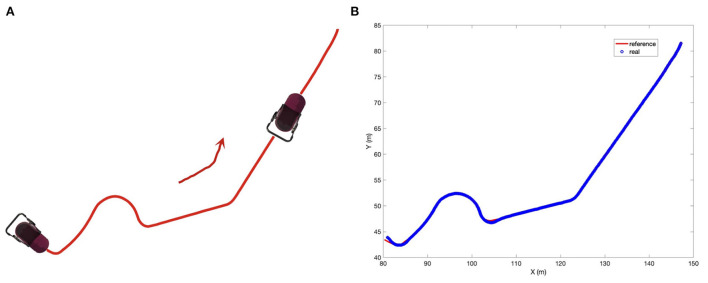
**(A)** The illustration of experimental setup for an arbitrary path tracking task for the baby stroller control. **(B)** Experimental results of following an arbitrary path.

**Figure 14:**
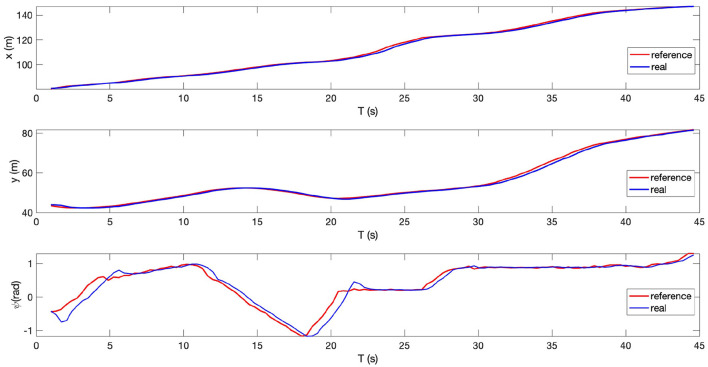
Longitudinal position, lateral position, and yaw angle of the reference trajectory and of the baby stroller when following an arbitrary path.

In real life, the real path may be an irregular curve with varying curvature. Therefore, in addition to the line tracking and circle tracking tasks, we further designed and performed an arbitrary path tracking task where the reference path was given in advance. The experimental setup is illustrated in Figure 13. As the other two tasks, the simulation is with MATLAB. The desired velocity is set at 2 *m*/*s*. The tracking performance can be seen from Figures 13, 14, where the red curves denote the given reference path while the blue circles/curves are the real stroller trajectory. From We see that the baby stroller follows the reference path well. The performance can be further observed from Figure 14, where the longitudinal, lateral positional and yaw angle of the reference and the real stroller trajectory are compared. The acceptable accuracy and smoothness validate the controller effectiveness. We hence conclude that the proposed MPC strategy is able to improve the efficiency of the four-wheeled baby stroller in common situations.

**Figure 15:**
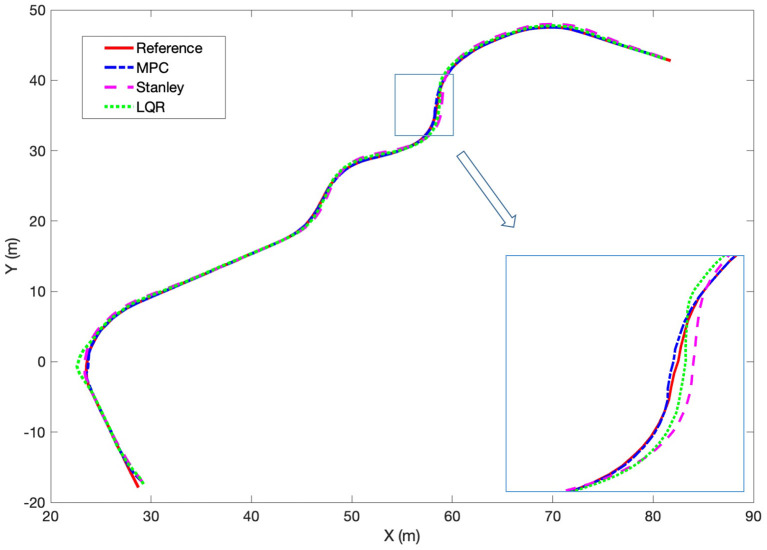
The comparison of the tracking performance of MPC, Stanley, and LQR approaches.

We carried out a contrastive experiment of an arbitrary path tracking task to further illustrate the effectiveness of our proposed controller. The model predictive control algorithm, the Stanley algorithm and the linear quadratic regulator (LQR) algorithm are used in simulation for comparison. The reference trajectory is given, containing both line segments and turns to represent the scenarios in the real world. The comparison results are shown in Figure 15. It can be observed that for line segments, all methods can track the reference well, while for turns, the MPC method outperforms the other two methods, with the best tracking accuracy. In the enlarged area, we can see that the deviation from the reference curve for MPC is almost zero, while the LQR method and Stanley method have deviation errors of 0.3 and 0.5 m, respectively. Obviously, the MPC controller has faster response, higher tracking accuracy and smoothness, satisfying the motion requirements of the baby stroller.

The four-wheeled baby stroller is an integrated application for assisting parents with newborn baby care. The design must take into consideration the practical demands of the users. More importantly, safety and stability are factors when it comes to controlling the stroller's motion. Although there are plenty of works on the lateral stability motion control in the literature, few studies concentrate on the control of the baby stroller, which is a main challenge of the commercial products. Therefore, we endeavor to design an active control of the baby stroller. For this aim, we have presented a lateral stability control scheme based on motion predictive control and verified its effectiveness through path tracking tasks in simulation. The good performance in terms of low tracking error and high smoothness meets the requirements of low-speed stability, safety and reliability, indicating that it has promising potential to provide practical assistance in the consumer market.

## 5. Conclusions and future work

The functionality of current existing baby stroller products can not satisfy the requirements. In order to open up new growth space for baby stroller design, this paper proposed a novel modularization design and safety-enhanced control scheme for empowering and assisting mobility. The main contributions of this work consist of:

1. We introduced a modularization design method to design a novel four-wheeled baby stroller featuring safety-enhanced movement posture control to cater to multifunctional demands.
2. We designed a lateral stability tracking controller based on the model predictive control scheme to control the motion of the baby stroller in a safe, stable, and smooth manner.

We believe that our work is beneficial to the increasing market demand for baby strollers. Currently, the validation of the stroller design and control is limited in the simulation environment. However, the real environment will be much more complex than simulation due to various factors such as sensor noise, uncertain disturbances, etc. This will pose great challenges to control. Therefore, based on the work we have done, in the future, we will further verify the design and motion control of the baby stroller in practical situation to ensure its robustness by carrying out more extensive and practical experiments on the actual system. Moreover, various challenging situations such as dead-zone and time-delay (Yang et al., 2016; Zhang et al., 2016; Luo et al., 2019a,b) shall be considered for the stroller being able to adapt to different environments, where system stability and tracking accuracy cannot be guaranteed. The stroller can stably and accurately track a given trajectory with the implemented MPC controller. However, how to generate the reference trajectory is another important yet challenging topic, especially in the uncertain environment with dynamic obstacles. Reference trajectory can be planned using global approaches such as rapidly-exploring random tree (RRT) or local methods like artificial potential field (APF). These traditional methods are limited to low-dimensional problems or prone to getting stuck in local minima. Recently, more and more researchers have resorted to reinforcement learning to tackle the complicated planning problems for uncertain environments with human coexistent (Chen et al., 2022). This will be a future research direction for the baby stroller autonomous movement. Finally, human-machine interaction (Li et al., 2014, 2015a; Yang et al., 2017; Su et al., 2019; Luo et al., 2021) is also an essential topic that will be further researched.

## Data availability statement

The raw data supporting the conclusions of this article will be made available by the authors, without undue reservation.

## Author contributions

CZ, ZH, and WS: conceptualization, investigation, and data curation. CZ, ZH, WS, and LD: methodology, software, validation, formal analysis, and writing—original draft preparation. CZ, ZH, XH, WS, and LD: writing—review and editing. All authors read and edited the manuscript, and agrees with its content. All authors contributed to the article and approved the submitted version.

## Funding

This study was supported in part by the planning subject for the 13th 5 year plan of Guangdong Education Sciences under Grand no. 2020GXJK335, in part by Guangdong Higher Education Teaching Reform Project under Grand no. SJY202002.

## Conflict of interest

The authors declare that the research was conducted in the absence of any commercial or financial relationships that could be construed as a potential conflict of interest.

## Publisher's note

All claims expressed in this article are solely those of the authors and do not necessarily represent those of their affiliated organizations, or those of the publisher, the editors and the reviewers. Any product that may be evaluated in this article, or claim that may be made by its manufacturer, is not guaranteed or endorsed by the publisher.
